# High antiviral effect of TiO_2_·PL–DNA nanocomposites targeted to conservative regions of (−)RNA and (+)RNA of influenza A virus in cell culture

**DOI:** 10.3762/bjnano.7.108

**Published:** 2016-08-10

**Authors:** Asya S Levina, Marina N Repkova, Elena V Bessudnova, Ekaterina I Filippova, Natalia A Mazurkova, Valentina F Zarytova

**Affiliations:** 1 Institute of Chemical Biology and Fundamental Medicine, Siberian Branch of Russian Academy of Sciences, pr. Lavrent’eva 8, Novosibirsk, 630090, Russia; 2 Institute of Catalysis, Siberian Branch of Russian Academy of Sciences, pr. Lavrent’eva 5, Novosibirsk, 630090, Russia,; 3 FBRI State Research Center of Virology and Biotechnology "Vector", Koltsovo, Novosibirsk region, 630559, Russia

**Keywords:** conservative regions, DNA fragments, H1N1, H5N1, and H3N2 subtypes of influenza A virus, TiO_2_·PL–DNA nanocomposites

## Abstract

**Background:** The development of new antiviral drugs based on nucleic acids is under scrutiny. An important problem in this aspect is to find the most vulnerable conservative regions in the viral genome as targets for the action of these agents. Another challenge is the development of an efficient system for their delivery into cells. To solve this problem, we proposed a TiO_2_·PL–DNA nanocomposite consisting of titanium dioxide nanoparticles and polylysine (PL)-containing oligonucleotides.

**Results:** The TiO_2_·PL–DNA nanocomposites bearing the DNA fragments targeted to different conservative regions of (−)RNA and (+)RNA of segment 5 of influenza A virus (IAV) were studied for their antiviral activity in MDCK cells infected with the H1N1, H5N1, and H3N2 virus subtypes. Within the negative strand of each of the studied strains, the efficiency of DNA fragments increased in the direction of its 3’-end. Thus, the DNA fragment aimed at the 3’-noncoding region of (−)RNA was the most efficient and inhibited the reproduction of different IAV subtypes by 3–4 orders of magnitude. Although to a lesser extent, the DNA fragments targeted at the AUG region of (+)RNA and the corresponding region of (−)RNA were also active. For all studied viral subtypes, the nanocomposites bearing the DNA fragments targeted to (−)RNA appeared to be more efficient than those containing fragments aimed at the corresponding (+)RNA regions.

**Conclusion:** The proposed TiO_2_·PL–DNA nanocomposites can be successfully used for highly efficient and site-specific inhibition of influenza A virus of different subtypes. Some patterns of localization of the most vulnerable regions in IAV segment 5 for the action of DNA-based drugs were found. The (−)RNA strand of IAV segment 5 appeared to be more sensitive as compared to (+)RNA.

## Introduction

The fundamental scientific problem of life sciences, especially modern molecular biology and fundamental medicine, is the development of approaches to the directed action on the genetic material of cells. Fragments of nucleic acids (oligonucleotides and their derivatives and analogs) can be used for this purpose because they are known to site specifically interact with certain regions of nucleic acid (NA) targets. The development of antisense technology for the creation of drugs that can gene specifically affect the genes responsible for infectious, hereditary, cancer, and cardiovascular diseases is an important task of modern medicine and pharmacology. The selective recognition of molecular targets by NA-based therapeutics may minimize negative side effects compared to conventional pharmaceuticals that typically have less specificity.

Influenza A viruses (IAVs) are prominent in infectious diseases of humans and animals and periodically cause epidemics and epizootics. At present, the development of new antiviral drugs based on native or chemically modified nucleic acids is under scrutiny. Researchers all over the world explore the therapeutic potential of NA-based drugs to fight against the genetically variable viruses including IAV.

Many attempts to produce effective antiviral drugs against IAV have not yet been successful because of the low ability of NA-based drugs to penetrate into cells. In the last few years, there were a large number of publications describing different delivery systems of nucleic acid fragments into cells [1–3], including the use of inorganic nanoparticles [4].

Another important problem of using NA-based drugs is to select a proper target in the IAV genome. The choice of the most efficient antiviral NA-based drugs to affect IAV replication may contribute to the creation of therapeutic preparations, the activity of which would be less dependent on mutations. The IAV genome contains eight single-stranded RNA segments of negative polarity and belongs to the Ortomyxoviridae family. All eight segments are important for the viral replication. After infection of cells, the viral (−)RNA segments are transcribed into (+)mRNA and replicated into (+)cRNA [5]. The IAV genome is known for its high variability. The most variable genes are segments 4 and 6, encoding hemagglutinin and neuraminidase, respectively. Segments 1, 2, 3, 5, and 7, encoding RNA-dependent RNA polymerases (PB2, PB1, and PA), nucleoprotein (NP), and matrix protein M1, respectively, comprise the largest number of conserved sequences [6]. The NP plays a key role in the migration of the viral RNAs to cell nuclei of infected cells and in the following replication and assembly of the virus [7]. Therefore, many researchers believe that segment 5, which encodes this protein, is an ideal target to block the virus reproduction by antiviral compounds [8–10].

Different regions of segment 5 have been studied for their sensitivity to the action of NA-based drugs of various nature [8–16]. However, there is still no unified concept for the most vulnerable regions of this segment. In this work, we attempt to find some relationships in this aspect for IAV segment 5.

To deliver antisense oligonucleotides into cells, we previously proposed the TiO_2_·PL–DNA system [17], where oligonucleotides were noncovalently immobilized on TiO_2_ nanoparticles through the polylysine (PL) linker. It was shown that TiO_2_·PL–DNA nanocomposites can penetrate into cells without any additional treatment (i.e., transfection agents or physical impact). It was demonstrated that these nanocomposites exhibited a low toxicity and very high activity against IAV in the cell culture [18–19]. Only one site in the IAV 5 segment was used in our previous works.

In this work, we examined the antiviral activity of DNA fragments in the proposed TiO_2_·PL–DNA nanocomposites, targeted to conservative regions of (−)RNA and (+)RNA of different IAV subtypes (H1N1, H5N1, and H3N2).

## Results and Discussion

The choice of the most suitable regions in nucleic acids for oligonucleotide-based agents is of great importance. In order to inhibit the different subtypes of viruses, these regions should have conservative nucleotide sequences and be accessible for the interaction with complementary oligonucleotides. As many other researchers have [8–16], we have chosen segment 5 (encoding nucleoprotein (NP)) of IAV genome as a target to affect the virus replication. To find the most conservative regions of segment 5, we analyzed nucleotide sequences of various subtypes of IAV available in the NCBI Influenza Virus Resource database (http://www.ncbi.nlm.nih.gov). We have chosen the four most conservative regions, i.e. the noncoding region located on the 3’-end of (+)cRNA after the stop codon (1544–1564), the nearby coding region close to the stop codon (1498–1516), the region including the AUG codon (34–54), and the 5’-noncoding region (2–22), where the nucleotide numbering is given for (+)cRNA. The short 5’- and 3’-terminal nontranslated regions (NTRs) are of considerable interest because all eight RNA segments of various IAV subtypes are known to contain conserved sequences of 13 or 12 nucleotides at their 5’ and 3’ ends (^5’^AGUAGAAACAAGG^3’^and ^5’^CCUGCUUUUGCU^3’^, respectively) [20–21].

The region including the AUG codon in (+)RNA [11,14] and the 3’-terminal region of (−)RNA [10–11] were the most chosen regions of IAV segment 5 to be affected by oligonucleotides and their analogs. The coding region close to the stop codon was often used as the target for the action of siRNA [9,12,15–16]. The other regions of the NP gene were also studied as targets [8,11–12,21]. It can be concluded from these literature data that the most susceptible regions for the action of NA-based drugs are the first two regions mentioned above. Nevertheless, a comprehensive study was not performed concerning the susceptibility of different regions of the IAV segment 5 to the action of NA-based compounds, which was attempted in the presented work.

**Figure 1:**
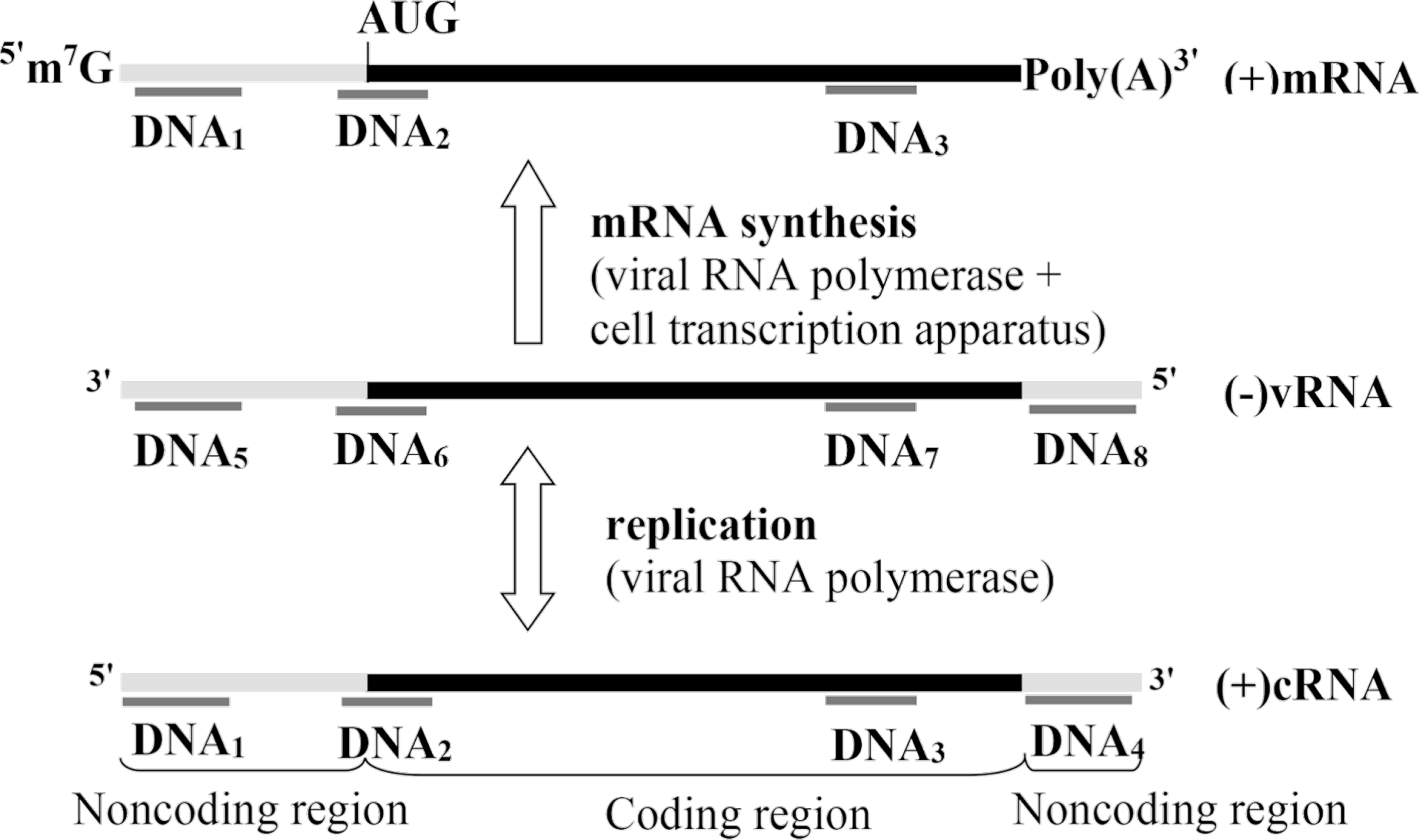
Scheme of localization of the used DNA fragments on influenza A virus segment 5. AUG is the initiation codon for translation of the nucleoprotein gene.

As mentioned above, after infection of cells viral (−)RNA are transcribed into (+)mRNA and replicated into complementary (+)cRNA [5]. To affect the viral genome, we used oligodeoxynucleotides directed to four selected conserved regions of the NP gene, which were complementary to both (−)RNA and (+)RNA (Table 1). The localization of these DNA fragments on viral RNA is depicted in Figure 1. The nucleotide sequences were chosen so that they had the terminal G/C nucleotides to provide more stable complexes with the target and did not form the hairpin and self-complementary structures. All oligonucleotides were 19–21-mers. In addition to these oligonucleotides, a random sequence (DNA-r) was used as a control.

**Table 1:** Oligonucleotides aimed at different regions of influenza A virus segment 5.

Sequence, 5’→3’	Name	Targeted at	Position in segment 5^a^

GATTATCTACCCTGCTTTTGCp	DNA_1_	5’-noncoding region of (+)RNA	2–22
GCAAAAGCAGGGTAGATAATCp^b^	DNA_5_	corresponding 3’-region of (−)RNA	
GAGACGCCATGATGTTGATGTCp	DNA_2_	AUG region of (+)RNA	34–54
GACATCAACATCATGGCGTCTCp	DNA_6_	corresponding region of (−)RNA	
CTCCGAAGAAATAAGATCCp	DNA_3_	3’-coding region of (+)RNA	1498–1516
GGATCTTATTTCTTCGGAGp	DNA_7_	corresponding 5’-region of (−)RNA	
GTAGAAACAAGGGTATTTTTCp	DNA_4_	3’-noncoding region of (+)RNA	1544–1564
GAAAAATACCCTTGTTTCTACp	DNA_8_	corresponding 5’-region of (−)RNA	
GATCAACTCCATATGCCATGTp	DNA_r_	random	–

^a^Numbering of nucleotides are given for (+)RNA; ^b^Nanocomposite TiO_2_·PL–DNA containing this DNA fragment was used in previous works [18–19].

The synthetic DNA fragments were conjugated with polylysine (PL), and the resultant PL–DNA conjugates were noncovalently immobilized on TiO_2_ nanoparticles (≈5 nm in diameter) in the anatase form [17] due to the affinity of polylysine to the TiO_2_ surface. This affinity can be explained in all likelihood by the electrostatic interaction between the positively charged amino groups of PL and the negatively charged TiO_2_ surface at neutral pH [22]. Since the PL–DNA conjugate contains a 100-fold excess of the amino groups over an oligonucleotide [17], the majority of the amino groups are free to interact with the negatively charged surface of the TiO_2_ nanoparticles.

The resultant TiO_2_·PL–DNA nanocomposite was used to inhibit the reproduction of IAV in cell culture. TiO_2_ nanoparticles (of ≈5 nm in diameter) are known to penetrate through cell membrane [23]. It was clearly demonstrated in our previous work [17–18] that they are good vehicles to transport DNA fragments into cells. There are literature data that show that nanoparticles protect oligonucleotides against nucleases [24]. We think that in our case TiO_2_ nanoparticles may also play a protective role. In addition, the PL linker attached to the 3’-end of the DNA fragment in PL–DNA also protects oligonucleotides from cellular nucleases. Thus, native DNA fragments with phosphodiester internucleotide bonds can be used without any additional modification.

We investigated the antiviral action of eight nanocomposites of this type bearing DNA fragments complementary to eight conservative regions of RNA segment 5 of negative and positive polarity (Figure 1, Table 1) of different IAV subtypes.

The inhibiting effect of the chosen DNA fragments on the virus reproduction was examined in MDCK cells infected with one of three different subtypes of influenza A virus (H1N1, H3N2, and H5N1).

**Figure 2:**
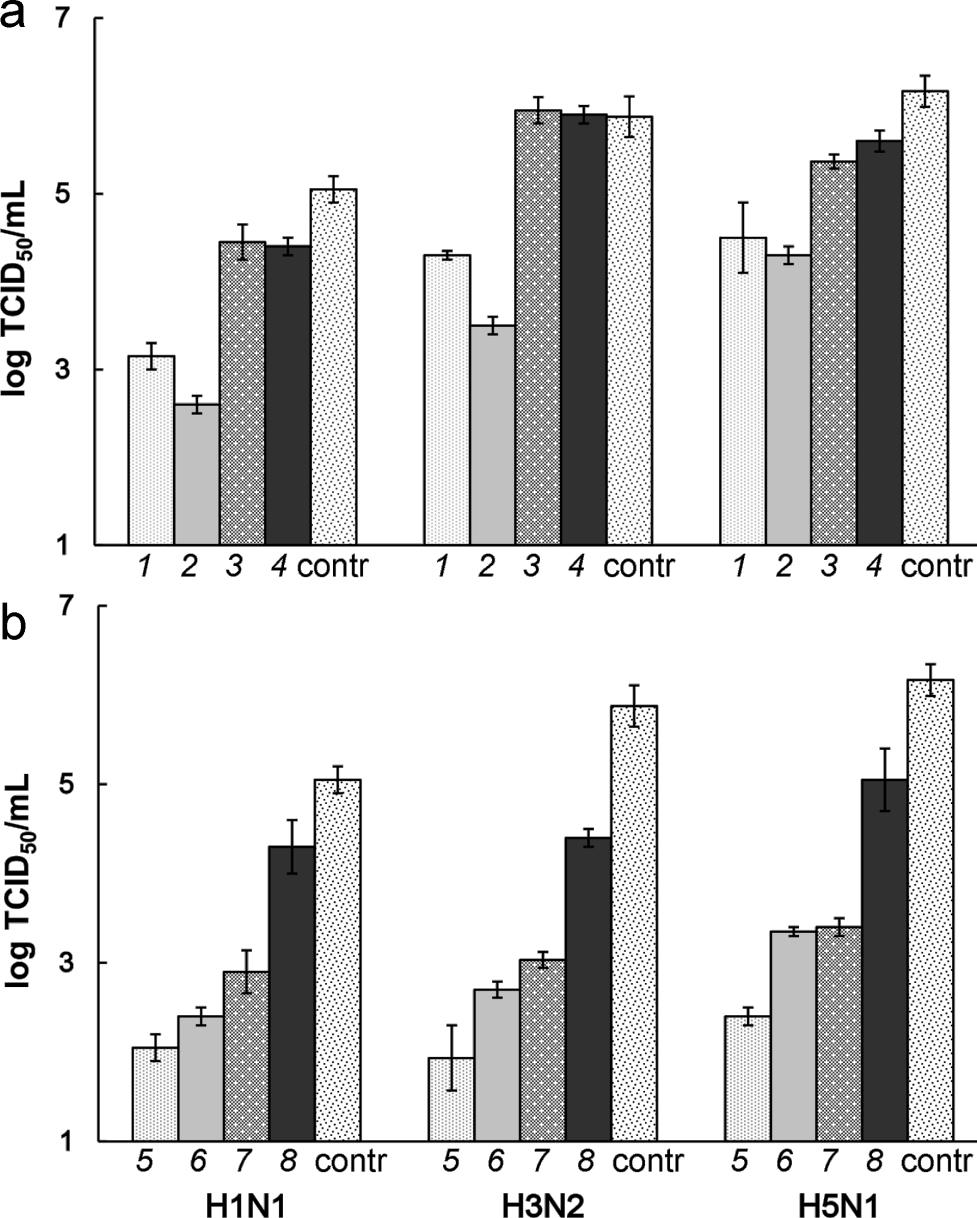
IAV titers in MDCK cells infected with H1N1, H3N2, and H5N1 IAV subtypes and treated with TiO_2_·PL–DNA nanocomposites containing DNA_1_–DNA_8_ fragments (columns 1–8, respectively). The location of these fragments on IAV strands can be seen in Figure 1. The nanoparticle concentration was 5 μg/mL and the DNA fragment concentration was 0.1 μM (20 nmol/mg). The values are mean values from three experiments. The results are given for the studied samples targeted to (a) (+)RNA and (b) (−)RNA.

The antiviral activity of the nanocomposites was studied in the postinfection assays, that is, the cells were initially infected with the virus and then incubated with nanocomposites. The experiments were carried out at a multiplicity of infection (MOI) of 0.1 TCID_50_/cell and at 5 µg/mL concentration of the nanocomposites, which corresponded to a 0.1 µM concentration for the DNA fragment. This nanocomposite concentration was much lower than TC_50_/mL (1800 µg/mL [18]). The results are presented in terms of the TCID_50_/mL values in Table 2. Table 2 also shows the extent of the IAV inhibition (*n*-fold) with the studied nanocomposites in comparison with the control without any samples. In addition, the results are demonstrated in Figure 2 in terms of logTCID_50_/mL.

**Table 2:** Antiviral activity of the studied TiO_2_·PL–DNA nanocomposites. Virus titers and inhibition of virus reproduction (*n*-fold) in MDCK cells infected with different IAV subtypes after treating with nanocomposites bearing DNA fragments complementary to four conservative regions of (−)RNA and (+)RNA of segment 5.

	Sample	Targeted at	H1N1		H3N2		H5N1	
			Virus titer	≈*n*-fold	Virus titer	≈*n*-fold	Virus titer	≈*n*-fold
			TCID_50_/mL		TCID_50_/mL		TCID_50_/mL	

1	TiO_2_·PL–DNA_1_	(+)RNA	1413	80	19953	38	31623	35
2	TiO_2_·PL–DNA_2_		398	300	3162	240	19953	60
3	TiO_2_·PL–DNA_3_		28184	4	891251	1	316228	4
4	TiO_2_·PL–DNA_4_		25119	4	794328	1	398107	3
5	TiO_2_·PL–DNA_5_	(−)RNA	112	1000	86	8900	251	4500
6	TiO_2_·PL–DNA_6_		251	460	501	1500	2239	500
7	TiO_2_·PL–DNA_7_		794	150	1080	700	2512	450
8	TiO_2_·PL–DNA_8_		19953	6	25119	30	112202	10
9	TiO_2_·PL–NA_r_	controls	15849	7	50119	15	199526	6
10	TiO_2_		–	–	702583	1	1037884	1
11	TiO_2_·PL		–	–	532753	1.4	781882	1.5
12	w/o sample	virus control	116591		762957		1141563	

First of all, it should be noted that the studied nanocomposites containing the DNA fragments targeted to (−)RNA of IAV segment 5 were more efficient against the virus as compared to those targeted to (+)RNA (compare rows 1–4 and 5–8 in Table 2 and columns of the same color in Figure 2a and Figure 2b). Three of the nanocomposites inhibited the virus replication by factors higher than 100 (rows 5–7 in Table 2 and columns 5–7 in Figure 2b) for all studied strains. The most pronounced difference (by 1–3 orders of magnitude) between the nanocomposites aimed at the (+)RNA and (−)RNA was detected for the DNA_1_/DNA_5_ pair (Table 2, row 1 vs row 5; Figure 2a,b, columns 1 vs columns 5) and DNA_3_/DNA_7_ pairs **(**Table 2, row 3 vs row 7; Figure 2, columns 3 vs columns 7) when one of the oligonucleotide of the pair showed either very high (DNA_5_) or very low (DNA_3_) activity. There is a small difference (if at all) between the counterparts when they both exhibit either the low or high activity (the DNA_4_/DNA_8_ and DNA_2_/DNA_6_ pairs, respectively).

It can be seen that the nanocomposites bearing the DNA fragments targeted to (−)RNA became more potent as they move along the (−)strand from the 5’ to the 3’ end (rows 8 to 5) (Figure 2b, columns DNA_8_ to DNA_5_**)**. Thus, the DNA_5_ fragment, which inhibited the virus reproduction by up to 3–4 orders of magnitude (row 5), appeared to be the most active while the DNA_8_ fragment targeted to the opposite 5’-end of (−)RNA (row 8) showed low activity.

Only one of the studied nanocomposites containing the DNA fragment complementary to (+)RNA was quite active (row 2, columns 2), which was TiO_2_·PL–DNA_2_ bearing the DNA fragment targeted to the AUG region of (+)RNA. And yet, the antiviral activity of this fragment was lower than that of the corresponding DNA fragment targeted to (−)RNA (compare rows 2 and 6 in Table 2, and columns 2 and 6 in Figure 2). The TiO_2_·PL–DNA_2_ nanocomposites are superior to the TiO_2_·PL–DNA_1_ nanocomposite **(**compare row 1 and 2, and columns 1 and 2) with regards to the antiviral effect, and the difference between DNA_4_ and DNA_5_ fragments is low (rows 4 and 5, columns 4 and 5). Thus, in the case of the (+)RNA strand, there is no regular increase or decrease in the activity of the DNA fragments as they move along this strand.

It is not surprising that for all studied subtypes, the difference between the nanocomposites bearing the site-directed DNA fragments and those containing the random sequence was significant. The maximal difference of 2–3 orders of magnitude was observed for the most efficient nanocomposite (TiO_2_·PL–DNA_5_, Table 2).

Summarizing the results, we can say that four of eight examined sites of (+)RNA and (−)RNA of IAV segment 5 were the most vulnerable to the action of the DNA fragments in the proposed nanocomposites. Specifically, the AUG region of (+)RNA and the corresponding region of (−)RNA, the region in (−)RNA complementary to the coding region in (+)RNA, and especially the 3’-end of (−)RNA (rows 2, 6, 7, and 5, respectively, in Table 2). In contrast, the 3’-noncoding and nearby coding regions of (+)RNA, as well as corresponding to the former the 5’-end of (−)RNA, were the least susceptible (if at all) to the antiviral action of the proposed nanocomposites (rows 4, 3, 8 and columns 4, 3, 8, respectively). Their activities were comparable with the action of the DNA fragment with a random sequence (Table 2, row 10).

The fact that the initial viral (−)RNA of IAV segment 5 is more vulnerable to the action of DNA fragments than the (+)strand may indicate that the negative strand is attacked immediately after its release from the complex with the viral proteins (nucleoprotein and polymerases) before transcription into (+)mRNA and replication into complementary (+)cRNA.

The most efficient DNA_5_ oligonucleotide directed to the 3’-noncoding region of (−)vRNA contains the conserved sequence of 12 nucleotides common for all eight RNA segments of various IAV strains. Most likely, the observed strong effect of this fragment is partially explained by its simultaneous action to the other segments of the viral genome.

It should be noted that the use of the proposed TiO_2_·PL–DNA nanocomposites leads to the more efficient inhibition of the IAV replication as compared to the data of other researchers who used NA-based agents against IAV segment 5 [8–16].

Transfection of the IAV-infected MDCK cells with phosphorothioate oligonucleotide (4 µM) targeted to the internal coding regions of the (+)strand of the NP gene (NP-267, NP-628, and NP-749) in the presence of lipofectamine led to a slight reduction of the virus titer by 0.93–1.23 log [8]. The DOTAP-mediated transfection of the clone 76 cells with phosphorothioate oligonucleotides directed to the AUG region of (+)RNA of different segments, including the NP segment, demonstrated a little more than 50% inhibition of IAV at 0.3 µM concentration [14]. Duan et al. [25] used a 13-mer phosphorothioate oligonucleotide directed to the 5’-terminal region of (−)RNA of segment 5 (the fragment of our DNA_8_). They showed that this DNA fragment was efficient against various strains of IAV. The best IC_50_ and SI_50_ values were 2.22 µM and 394, respectively. Unfortunately, the authors did not indicate the level of the virus inhibition.

Morpholino oligonucleotides with the same sequences as our DNA_5_, DNA_1_, DNA_8_, and DNA_4_ were examined against the NP segment [10]. To ensure the delivery into cells, morpholino oligonucleotides were conjugated to an arginine-rich peptide. The efficiency of the used oligomers were in the following order: DNA_1_ << DNA_8_ < DNA_4_ ≤ DNA_5_ (reduction by 50% or 2-fold, 75% or 4-fold, 85% or 7-fold, and 98% or 50-fold, respectively, at 5 µM concentration). This order differed from our case (DNA_4_ << DNA_1_ ≈ DNA_8_ ≤ DNA_5_) but the most efficient DNA fragment was the same, although its efficiency in the virus inhibition was still significantly lower even at higher concentration than in our case (3–4 orders of magnitude at 0.1 µM concentration). Morpholino oligonucleotides targeted at the NP gene with the sequence as our DNA_5_ and DNA_2_ fragments showed high inhibitory activity in cells infected by influenza A H7N7 virus (the viral replication was reduced by 3 and 6 log TCID_50_/mL, respectively, at a MOI of 0.001 and 5 µM concentration of oligomers) [11]. The DNA_4_ morpholino oligonucleotide displayed very low activity in these experiments. It was shown [21] that a 12-mer oligonucleotide directed to the 3’-end of (−)RNA of all IAV segments and delivered into cells with lipofectamine inhibited the IAV replication by 60% (2.5-fold) at a MOI of 0.1 and 5 nM concentration.

The above literature data confirm the idea about the usefulness of NA-based agents as antiviral drugs. Our results along with the literature data show that the 3’-nontranslated and AUG-containing conserved regions are most vulnerable for the antiviral action of NA-based compounds.

It should be emphasized that our experiments were carried out at a relatively high infection of cells (0.1 MOI) and at a low concentration of unmodified DNA fragments (0.1 µM) in the nanocomposites. The most active DNA_5_ fragment inhibited the reproduction of virus of the H1N1, H5N1, and H3N2 strains by factors of ≈1000, ≈3500, and ≈9000, respectively (Table 2), which was much higher than the effect of DNA fragments described in literature. The working concentration of the nanocomposites (5 µg/mL) was much lower than the TC_50_/mL value (1800 µg/mL) [18]).

## Conclusion

For the effective delivery of oligonucleotides into cells, we used TiO_2_·PL–DNA nanocomposites capable of penetrating without transfection reagents or physical impact. DNA fragments in the TiO_2_·PL–DNA nanocomposites were targeted to conservative regions of (−)RNA and (+)RNA of influenza A virus of the H1N1, H5N1, and H3N2 subtypes.

We found some patterns of localization of the most vulnerable regions in IAV segment 5 for the action of DNA-based drugs, where these patterns were the same for all tested subtypes. The (−)RNA of segment 5 appeared to be a more vulnerable strand of IAV as compared to the corresponding (+)RNA strand. The most sensitive sites in the IAV segment 5 to the action of the proposed agents were found to be the AUG-containing site in (+) RNA, the corresponding region in (−)RNA, and especially the 3’-noncoding region of (−)RNA. The most efficient TiO_2_·PL–DNA_5_ nanocomposite was highly active against all three strains (H1N1, H5N1, and H3N2), with the H3N2 strain being the most susceptible to the antiviral effect of the proposed nanocomposites. The results of this work may be taken into account when choosing suitable targets in the IAV genome for the action of nucleic acid-based drugs.

It should be noted that the effect of the most efficient nanocomposites on the viral reproduction (inhibition of the IAV replication by 3–4 orders of magnitude) was higher than that of other NA-based compounds described in literature (especially if the conditions used are taken into account, i.e., concentration of compounds and multiplicity of infection). It is important to emphasize that we used the most affordable, inexpensive, unprotected phosphodiester oligodeoxynucleotides.

Owing to their remarkable antiviral activity, the proposed TiO_2_·PL–DNA nanocomposites offer great potential to serve as platforms for drug development against a broad array of diseases involving nucleic acids that spans from infectious diseases to hereditary disorders.

## Experimental

### Materials and methods

All chemicals were obtained from commercial suppliers: RPMI-1640 medium; antibiotics (BioloT, Russia); trypsin, L-glutamine; PBS buffer (Sigma, USA); and fetal calf serum (Gibco, USA). TiO_2_ nanoparticles were synthesized in the crystal form (anatase) as described in [17]. Сhicken erythrocytes, MDCK cells, and influenza A virus strains Aichi/2/68 (H3N2), A/chicken/Kurgan/05/2005(H5N1), and А/Salekhard/01/2009 (H1N1) were from FBRI Vector, Russia. Trypsin (1 mg/mL) and penicillin-streptomycin (100 U/mL) (Sigma-Aldrich, USA) were stored at −80 °C. The IAV strains were grown in the allantoic cavity of 10-day-old embryonated chicken eggs at 37 °C. Allantoic fluid was harvested for 48 h after virus inoculation, aliquoted, and stored at −80 °C.

### Synthesis of nanocomposites

Oligonucleotides were synthesized by the phosphoramidite method on an ASM-800 DNA synthesizer (Biosset, Russia) using phosphoramidite monomers (Glen Research, USA). The polylysine (PL)-containing oligonucleotides (PL–DNA) were synthesized as described in [17]. The TiO_2_·PL–DNA nanocomposites were prepared by mixing PL–DNA and anatase nanoparticles (≈5 nm diameter) at room temperature for 20–30 min. The yield of the immobilization was ≈95%, with the capacity of the nanocomposites for oligonucleotides being ≈20 nmol/mg. A more detailed description of the nanocomposite preparation can be found elesewhere [17].

### Antiviral activity of nanocomposites

The MDCK cells in logarithmic phase were seeded at 100,000 cells/mL in RPMI-1640 nutrient medium containing 10% fetal calf serum (Gibco, USA) in 96-well plates (100 μL/well) and incubated at 37 °С, 5% CO_2_, and 100% humidity. The cells at ≈80% confluence were initially infected with one of the IAV subtypes, which was added into each well in RPMI-1640 medium (100 μL) containing trypsin (2 μg/mL) at a multiple infection of 0.1 TCID_50_/cell. The control sample was RPMI-1640 medium (100 μL) containing trypsin (2 μg/mL). After 1 h of virus adsorption at room temperature, the virus-containing medium was removed, and the cells were rinsed with RPMI-1640 medium without trypsin. The studied samples of the nanocomposite taken in RPMI-1640 medium without trypsin (100 μL/well) at a concentration of 5 μg/mL were applied to the infected MDCK cells, followed by incubation for 4 h at 37 °С, 5% CO_2_, and 100% humidity. After incubation for 4 h at room temperature, the medium containing the sample was removed, the cells were rinsed with RPMI-1640 medium without trypsin, and the same medium containing trypsin was added into each well (100 μL). After incubation for 48 h, serial 10-fold dilutions (from 10^−1^ to 10^−8^) of the culture virus-containing liquid from each well were applied to MDCK cells for 48 h to evaluate the virus titer. The presence of the virus was visually determined under a microscope by the cytopathic action and in the hemagglutination reaction with a 1% suspension of chicken erythrocytes. The virus titer was expressed in terms of TCID_50_/mL or log TCID_50_/mL (Table 2 and Figure 2, respectively). The titer was evaluated by noting the highest dilution of the virus that caused the hemagglutination reaction.
